# Supplementary material for: High-resolution sweep metagenomics using fast probabilistic inference
Source: Wellcome Open Res. 2021 Oct 8;5:14. Originally published 2020 Jan 30. [Version 2] doi: 10.12688/wellcomeopenres.15639.2 (PMC8543175; doi:10.12688/wellcomeopenres.15639.2)
Supplement: Peer review history

# Peer review: Reviewer response for version 2

**DOI:** 10.21956/wellcomeopenres.19077.r46349

**Published:** 2021-10-22

**Authors:** Mahendra Mariadassou

**Affiliations:** 1 INRAE, MaIAGE, Université Paris-Saclay, Jouy-en-Josas, France

The authors answered all comments I raised and I have no further ones.

Is the rationale for developing the new method (or application) clearly explained?

Yes

Is the description of the method technically sound?

Yes

Are the conclusions about the method and its performance adequately supported by the findings presented in the article?

Partly

If any results are presented, are all the source data underlying the results available to ensure full reproducibility?

Yes

Are sufficient details provided to allow replication of the method development and its use by others?

Yes

Reviewer Expertise:

Statistics, Metagenomics.

I confirm that I have read this submission and believe that I have an appropriate level of expertise to confirm that it is of an acceptable scientific standard.

# Peer review: Reviewer response for version 1

**DOI:** 10.21956/wellcomeopenres.17135.r39845

**Published:** 2021-03-05

**Authors:** Mahendra Mariadassou

**Affiliations:** 1 INRAE, MaIAGE, Université Paris-Saclay, Jouy-en-Josas, France

The article introduces a new method for typing of metagenomics data that goes beyond the species level (whenever) and down to the lineage or strain level. The approach does not apply to WGS datasets but to datasets sampled from plate sweeps, where the complexity of the community has been massively reduced by growth medium selection and the focus in on strain resolution. 

 The work is well written and easy to understand but some limitations should be pointed out:

- The work is most relevant for well studied bacteria (like pathogens) for which there are good genome reference databases.
- The comparison with metakallisto is slightly unfair, as pointed out by the authors, since it tries to tackle the more complex job of strain (rather than lineage) identification. A fairer comparison would be to pool the strains into the same group as mSWEEP and assess how it performs for those groups. This is especially important as the groups used in mSWEEP are well separated (see Fig. 3 t-SNE plot) and thus the task of assigning single-colony isolates to those groups is much easier than assigning them to strains. 
- The synthetic mixtures are not very adverse to the methods: 3 samples from 3 lineages with abundances in the mix higher than 20% is a very simple mix. The Vietnamese example already correspond to more complex mixes (~10 lineages / sample) and it would have been nice to stress mSWEEP under those conditions.
- The noise level of 0.016 used as detection threshold would prevent the detection / quantification of rare lineages and means that whole plates can't be mixed if they encompass many lineages with abundances spanning several orders of magnitude. This appears to be the case for the *K. pneumionae *dataset. 

How does the method compare to other strain identification tools (like DUDes ^1^) that are not specific to plate sweeps? I expect the reduction in complexity induced by plate sweeps to benefit mSWEEP but it would be useful to prove it. Finally, how does the method behave when the single-colony isolate from the sweep is very far from all lineages / groups in the database? Does mSWEEP then fail with an informative message / warning?

Is the rationale for developing the new method (or application) clearly explained?

Yes

Is the description of the method technically sound?

Yes

Are the conclusions about the method and its performance adequately supported by the findings presented in the article?

Partly

If any results are presented, are all the source data underlying the results available to ensure full reproducibility?

Yes

Are sufficient details provided to allow replication of the method development and its use by others?

Yes

Reviewer Expertise:

Statistics, Metagenomics.

I confirm that I have read this submission and believe that I have an appropriate level of expertise to confirm that it is of an acceptable scientific standard, however I have significant reservations, as outlined above.

## References

[1] : DUDes: a top-down taxonomic profiler for metagenomics. *Bioinformatics* .2016;32(15) : 10.1093/bioinformatics/btw150 2272-2280 10.1093/bioinformatics/btw150 27153591

# Response

**Published:** 2021-09-29

**Authors:** Tommi Mäklin

**Affiliations:** University of Helsinki, Finland

We have thoroughly utilized the excellent feedback provided by both reviewers and made adjustments to our manuscript based on their recommendations and suggestions. Notably, we have added a new synthetic experiment assessing the performance of mSWEEP under conditions more resembling those found in the Vietnamese E. coli sequencing data as suggested by Mahendra Mariadassou. Included below is a point-by-point response to the concerns raised by both reviewers. We would also like to thank both reviewers for their time and outstanding feedback that has enabled us to improve the quality of our manuscript.

**Review 2 — Mahendra Mariadassou**

**Mahendra Mariadassou:**

*The work is most relevant for well studied bacteria (like pathogens) for which there are good genome reference databases.*

**Author reply:**

 We have made it more clear in the revised manuscript that the intended application of our method is for extensively studied bacteria that have readily available reference sequences by including the following sentence in the Discussion section of our manuscript:

*As with any method intended to identify sequence variation, the target species need to be relatively well known to allow building of sufficiently informative reference databases. This means that mSWEEP is most relevant for extensively studied bacteria, such as pathogens, and has only limited applications when working with samples containing high numbers of novel or uncharacterized species.*

 Thank you for pointing out the potential for a misunderstanding.

**Mahendra Mariadassou:**

*The comparison with metakallisto is slightly unfair, as pointed out by the authors, since it tries to tackle the more complex job of strain (rather than lineage) identification. A fairer comparison would be to pool the strains into the same group as mSWEEP and assess how it performs for those groups. This is especially important as the groups used in mSWEEP are well separated (see Fig. 3 t-SNE plot) and thus the task of assigning single-colony isolates to those groups is much easier than assigning them to strains.*

**Author reply:**

 We agree that the comparison is slightly unfair since the methods are designed to solve different, yet related, problems. In the revised manuscript we have addressed this issue by adding a supplementary figure (Extended Data Figure 1 in the new manuscript), where the estimates from metakallisto for the sequences within the lineages have been pooled together by summing them up. While pooling improves the performance of metakallisto, the method still does not perform as well as mSWEEP, demonstrating that the probabilistic model leveraged by mSWEEP is necessary for resolving lineage-level differences, and that the conclusions in our manuscript remain largely the same.

**Mahendra Mariadassou:**

*The synthetic mixtures are not very adverse to the methods: 3 samples from 3 lineages with abundances in the mix higher than 20% is a very simple mix. The Vietnamese example already correspond to more complex mixes (~10 lineages / sample) and it would have been nice to stress mSWEEP under those conditions.*

**Author reply:**

 We thank the reviewer for this suggestion and have accordingly added a new set of experiments, where 10 different *E. coli* lineages per sample are mixed at varying sequencing depths, resulting in coverages ranging between 50x to 0.10x. Although the new experiments are more complex, the accuracy of the results (Figure 5 in the revised manuscript) remain largely the same and, in fact, support the conclusion derived from the existing set of synthetic mixtures that mSWEEP performs well up to around the 0.016 relative abundance (corresponding to about 1.6x coverage), which was the background noise level we previously established.

**Mahendra Mariadassou:**

*The noise level of 0.016 used as detection threshold would prevent the detection / quantification of rare lineages and means that whole plates can't be mixed if they encompass many lineages with abundances spanning several orders of magnitude. This appears to be the case for the K. pneumionae dataset.*

**Author reply:**

 The noise level of 0.016 is specific to the sequencing depth that was used to generate the synthetic mixtures. Since each mixture contains 1 000 000 reads that are 100 bases long, a relative abundance of under 0.016 would correspond to less than 16 000 reads and <0.65x sequencing depth (the exact value depending on the organism) in the species that we investigated. Considering that the differences between the lineages can be quite minimal, identification at these sequencing depths will naturally be less reliable and depend heavily upon the quality and nature of the reference sequences. Due to these factors, some sort of thresholding is, in our opinion, necessary.

 Presumably the sequencing depth could be increased in order to better identify rare lineages but our data unfortunately did not provide grounds to evaluate mSWEEP in this kind of setting. Nevertheless, there is some preliminary support for this argument in the revised manuscript, where the complex synthetic mixtures contained approximately 5 times more reads than the experiments the noise level was derived from, and consequently we were able to recover lineages at 0.0156 relative abundance (1.56x sequencing depth) and even a few of the lineages at 0.0078 relative abundance (0.78x sequencing depth). However, in general we do not expect mSWEEP to be able to separate lineages with < 1x sequencing depth unless they are far from each other in terms of genetic distance.

 In any case, we thank the reviewer for pointing this issue out and have clarified the implications of the noise level in the manuscript with the following changes to the section where the noise level is mentioned:

*The results indicate that above this relatively low background noise level of 0.016, quantifying mixture samples is not expected to produce more false positive results than would be obtained from single-colony samples. In the synthetic mixtures, the observed background noise level corresponds to sequencing depths of around 0.30x (E. coli and K. pneumoniae) and 0.65x (S. epidermidis), which provides the bare minimum sequencing depth required to distinguish between the lineages of each species in samples with similar read lengths and sequencing depth.*

**Mahendra Mariadassou:**

*How does the method compare to other strain identification tools (like DUDes) that are not specific to plate sweeps? I expect the reduction in complexity induced by plate sweeps to benefit mSWEEP but it would be useful to prove it.*

**Author reply:**

 We thank you for pointing out other relevant strain identification tools. However, we would like to point out that our tool is designed to be used with large, bespoke reference databases with the goal of leveraging detailed information about strain-level variation in identification. This approach necessitates replacing the traditional alignment methods, such as bowtie2 used by for example DUDes and many other strain identification tools, with pseudoalignment to achieve practical runtimes.

 Additionally, DUDes specifically utilizes a version of the NCBI taxonomy standard that is no longer in use, which restricts the applications of the method. The author points out this issue, as well as the scaling issue in bowtie2 alignment, in their  doctoral dissertation ( http://dx.doi.org/10.17169/refubium-1123), published after the DUDes paper. To our knowledge, and based on the DUDes GitHub repository, DUDes has not been updated to address these issues nor, in fact, been maintained after its initial publication. In our opinion a comparison with mSWEEP is thus unwarranted and would likely add little of value to the comparisons already presented in our manuscript.

 Moreover, other strain identification tools that make use of curated databases, such as subsets of the NCBI RefSeq database, are unsuited to analysing strain and lineage-level variation in clinical sequencing data due to the fact that for many of the strains the correct — both in-time and geographically — reference sequence is simply not necessarily included in the database. These types of databases are designed to provide a general overview of representative high-quality sequences for the species rather than something representative of the possibly highly localized variation, which renders their application beyond species-level identification conditional on the samples containing only distantly related strains. Although some methods do offer means to use customized reference databases, they are typically designed to be run with the precompiled databases and may not extend well when stretched beyond their intended purposes to the types of application that we have designed mSWEEP for.

**Mahendra Mariadassou:**

*Finally, how does the method behave when the single-colony isolate from the sweep is very far from all lineages / groups in the database? Does mSWEEP then fail with an informative message / warning?*

**Author reply:**

 While this is truly a limitation of our method that we did not directly tackle, as also pointed out by Fabiano L. Thompson in the review, there fortunately has been further work in solving this exact limitation. In particular, a computational tool ( https://github.com/harry-thorpe/demix_check) has been developed  to check whether the contents of a mixed sample correspond to a related reference sequence, or sequences from the same lineage, in mSWEEP's reference database, and produce results that can be used to verify the fact. For further explanation, we would like to refer to our answer to Fabiano L. Thompson to the similar question about the presence of a novel bacteria in the analysed sample. We have rewritten a part of our discussion section to address this concern, with the relevant paragraph now reading as:

*As with any method intended to identify sequence variation, the target species need to be relatively well known to allow building of sufficiently informative reference databases. Similarly, to allow for sensible and easily interpretable inferences, the biological clustering of the reference database should be based on well-established biological entities, such as multi-locus sequence types (STs) or clonal complexes (CCs) which are frequently employed as labels of lineages. These limitations suggest that mSWEEP is most relevant for extensively studied bacteria, such as pathogens, and has only limited applications when working with samples containing high numbers of novel or uncharacterized species. However, following the introduction of mSWEEP, a separate computational tool has been developed to assess the suitability of the reference sequences to specific samples and to identify cases where mSWEEP struggles due to the presence of reads originating from novel genome sequences (https://github.com/harry-thorpe/demix_check). Consequently mSWEEP can also be used to estimate the quality of the reference collection and to discard contaminated or mis-identified genomes.*

# Peer review: Reviewer response for version 1

**DOI:** 10.21956/wellcomeopenres.17135.r37996

**Published:** 2020-03-16

**Authors:** Fabiano L. Thompson

**Affiliations:** 1 Laboratory of Microbiology, Institute of Biology, Federal University of Rio de Janeiro, Rio de Janeiro, Brazil

Maklin *et al*. propose a new tool for the study of lineages and strains obtained from metagenomics. First of all, congratulations for this excellent study.

 Indeed, bioinformatics tools to tap into the microbial diversity beyond genus and species level using metagenomics are limited. And particularly for those approaches involving an initial culture/plate medium step. The authors have closed this gap with mSWEEP but they have not taken advantage of this to mention in their title that the novel tool addresses strain/lineage diversity). Mathematical modelling, including maximum likelihood and binomial distances were used and formulas were shown. The author studied samples from a group of infected children to validate their system. They used MacConkey plates to obtain bacterial cells for metagenomics. Reference data comprised Campylobacter, Escherichia, Klebsiella, Staphylococcus. False positives were estimated and in general were below 0.3 (Fig 4; y axis %?). mSWEEP output is depicted in the frame of clonal complexes (CC; Figs 3, 7) which is useful in the context of previous studies on population structure of pathogens.

 Some minor remarks to be incorporated in a revised version:

- Seven formulas were provided. However, the elements of each of these formulas were not explained. Please include an explanation in the new version.
- The Hiseq sequencing coverage (and read numbers) were not informed. How many ilumina reads does one need to use mSWEEP with confidence?
- Also another limitation is the need to select a reference genome for comparison. The sample may contain a novel genome, a novel pathogen. How does the system handle such possible situation?
- It is not clear why certain CC, eg. *Escherichia coli* CC405 and CC95 are connected to *Klebsiella pneumoniae* CC36, CC45 (Fig 7). This figure seems rather mixed in taxonomic terms. Is this a result of co-occurrence?
- It seems to me that relevant references need to be considered in the revised final version as they address similar questions. Arevalo *et al.* (2019) ^1^ and Bobay *et al*. (2018) ^2^. 

Is the rationale for developing the new method (or application) clearly explained?

Yes

Is the description of the method technically sound?

Yes

Are the conclusions about the method and its performance adequately supported by the findings presented in the article?

Yes

If any results are presented, are all the source data underlying the results available to ensure full reproducibility?

Yes

Are sufficient details provided to allow replication of the method development and its use by others?

Yes

Reviewer Expertise:

Microbiology.

I confirm that I have read this submission and believe that I have an appropriate level of expertise to confirm that it is of an acceptable scientific standard.

## References

[1] : A Reverse Ecology Approach Based on a Biological Definition of Microbial Populations. *Cell* .2019;178(4) : 10.1016/j.cell.2019.06.033 820-834.e14 10.1016/j.cell.2019.06.033 31398339

[2] : ConSpeciFix: classifying prokaryotic species based on gene flow. *Bioinformatics* .2018;34(21) : 10.1093/bioinformatics/bty400 3738-3740 10.1093/bioinformatics/bty400 PMC619885529771275

# Response

**Published:** 2021-09-29

**Authors:** Tommi Mäklin

**Affiliations:** University of Helsinki, Finland

We have thoroughly utilized the excellent feedback provided by both reviewers and made adjustments to our manuscript based on their recommendations and suggestions. Notably, we have added a new synthetic experiment assessing the performance of mSWEEP under conditions more resembling those found in the Vietnamese E. coli sequencing data as suggested by Mahendra Mariadassou. Included below is a point-by-point response to the concerns raised by both reviewers. We would also like to thank both reviewers for their time and outstanding feedback that has enabled us to improve the quality of our manuscript.

**Review 1 — Fabiano L. Thompson**

**Fabiano L. Thompson:**

*Seven formulas were provided. However, the elements of each of these formulas were not explained. Please include an explanation in the new version.*

**Author reply:**

 We thank the reviewer for pointing out the issue of missing explanations and have revised the manuscript so that all elements are explained close to the formulas where they are used.

**Fabiano L. Thompson:**

*The Hiseq sequencing coverage (and read numbers) were not informed. How many ilumina reads does one need to use mSWEEP with confidence?*

**Author reply:**

 While mSWEEP does not by itself require a specific number of Illumina reads to work, some practical guidelines can be derived based on the detection threshold analysis presented in the paper. In our analyses related to Extended Data Figures S3 and S4, we found that the minimum relative abundance required to accurately distinguish between strains of the same species in the same sample (“background noise level” in our manuscript) was 0.016 in synthetic samples containing 1 000 000 reads that were 100bp long. This translates to a minimum requirement of at least 16 000 reads from a specific lineage, corresponding to a coverage of about 0.30x on an average *E. coli* or *K. pneumoniae* genome and 0.65x on an *S. epidermidis* genome. Further down in the manuscript we define the detection thresholds to be either the background noise level or the value obtained from the threshold construction process, whichever of the two is higher, which results in lineage-specific thresholds that could be analysed in a similar way as the background noise level.

 Unfortunately, the results from this type of analysis are specific to the samples and reference sequences in question and do not generalize to different types of data. However, by replicating this type of analysis on one’s own prospective set of reference sequences, similar coverage and read number requirements could be derived before sequencing any mixed samples, providing some guidelines as to how many reads are required for mSWEEP to work accurately. The detection threshold analysis does additionally provide confidence scores for the abundance estimates exceeding the thresholds, which can be used to assess the reliability of the results.

 We have expanded our Discussion section to include a remark on the possibility of using the detection thresholds to assess the required sequencing coverage by including the following statement:

*This pipeline also estimates the relative abundance of lineages and provides means to construct reliability cut-offs with accompanying confidence scores. Since the cut-offs are constructed before analysing any sequencing data by synthetically mimicking the properties of the in vitro data, the cut-offs and confidence scores can be used to assess the necessary sequencing depth to identify rare and low abundance lineages with confidence as well as the total number of reads required.*

 We further thank the reviewer for providing us the opportunity to better our manuscript by explaining this feature of our pipeline in more detail.

**Fabiano L. Thompson:**

*Also another limitation is the need to select a reference genome for comparison. The sample may contain a novel genome, a novel pathogen. How does the system handle such possible situation?*

**Author reply:**

 As pointed out by the reviewer and also in Mahendra Mariadassou's review, the absence of a reference sequence for a novel bacterial strain or species is absolutely a limitation of our method. In cases where there is no related genome at all in the reference genomes, a very low percentage of the reads will pseudoalign to the reference sequences at all, interrupting the analysis. In cases where the novel genome is related to the reference genomes (either a new lineage of an existing species or a closely related novel species), our method will naturally fail to identify the correct lineage. Based on our observations, the relative abundance estimates resulting from this scenario will be spread between the most closely related genomes that are contained in the reference which can, in some cases, produce results that appear deceptively similar to those from a real mixed sample. 

 While our method does not address this concern — other than indirectly in some specific cases through the detection threshold analysis described in the manuscript — there have been excellent further developments of our method in solving this exact problem after the manuscript was published. In particular, a supplementary computational method ( https://github.com/harry-thorpe/demix_check) has been developed for assessing whether the reference sequences contain representative sequences for the strains present in a (mixed) sample that has been processed by mSWEEP. We believe this tool neatly solves this particular limitation of our method and provides researchers an additional tool to properly judge whether the results of their analyses are correct or contain errors related to the presence of novel strains. We have rewritten a part of our discussion section to take into account the limitations pointed out by the reviewer and include our suggested solution. The relevant paragraph has been changed to the following:

*As with any method intended to identify sequence variation, the target species need to be relatively well known to allow building of sufficiently informative reference databases. Similarly, to allow for sensible and easily interpretable inferences, the biological clustering of the reference database should be based on well-established biological entities, such as multi-locus sequence types (STs) or clonal complexes (CCs) which are frequently employed as labels of lineages. These limitations suggest that mSWEEP is most relevant for extensively studied bacteria, such as pathogens, and has only limited applications when working with samples containing high numbers of novel or uncharacterized species. However, following the introduction of mSWEEP, computational tools have been developed to assess the suitability of the reference sequences to specific samples and to identify cases where mSWEEP struggles due to the presence of reads originating from novel genome sequences (https://github.com/harry-thorpe/demix_check). Consequently mSWEEP can also be used to estimate the quality of the reference collection and to discard contaminated or mis-identified genomes.*

**Fabiano L. Thompson:**

*It is not clear why certain CC, eg. Escherichia coli CC405 and CC95 are connected to Klebsiella pneumoniae CC36, CC45 (Fig 7). This figure seems rather mixed in taxonomic terms. Is this a result of co-occurrence?*

**Author reply:**

 Indeed, the connections between the *E. coli* and *K. pneumoniae* clonal complexes are a result of co-occurrence in the samples. These samples were specifically selected for our study because they had been identified as contaminated or containing mixed species or strains during the culture step in another study aimed at isolating *K. pneumoniae* strains. As a result, many of the samples presented in our study are taxonomically diverse and contain several different species, or the strains of, that also grow on the types of plates that were used to culture *K. pneumoniae*. We thank the reviewer for pointing out the confusing parts in our text and have clarified the reasons for the co-occurrence in the text with the addition of the following sentence:

*Since both E. coli and A. baumanii grow on the media used for culture of K. pneumoniae, frequent co-occurrence in samples selected for their diversity is expected.*

**Fabiano L. Thompson:**

 It seems to me that relevant references need to be considered in the revised final version as they address similar questions. Arevalo et al. (2019) and Bobay et al. (2018).

**Author reply:**

 We thank you for referring us to these highly relevant studies that we missed in our literature review and have addressed the potential benefits of combining gene flow based approaches with mSWEEP analyses in the discussion section of our revised version of the manuscript.
